# Biodegradable Films Fabricated by Calcium Cross-Linking of Walnut Protein Isolate and Cellulose Nanofiber with Incorporation of Curcumin for Strawberry Packaging

**DOI:** 10.3390/foods15152721

**Published:** 2026-08-02

**Authors:** Kunli Zhao, Bing Zhao, Yubo Zhu, Jia Luo, Jun Sheng, Yang Tian, Xiufen Li

**Affiliations:** 1 College of Food Science and Technology, Yunnan Agricultural University, 425 Fengyuan Road, Kunming 650201, China; zhaokunli2026@163.com (K.Z.); 2021008@ynau.edu.cn (B.Z.); zhuzhuyb15@163.com (Y.Z.); shengjun_ynau@163.com (J.S.); tianyang1208@163.com (Y.T.); 2 Kunming Branch, CAS Key Laboratory of Tropical Plant Resource and Sustainable Use, Xishuangbanna Tropical Botanical Garden, Chinese Academy of Sciences, 88 Xuefu Road, Kunming 650223, China; luojia@xtbg.ac.cn; 3 Engineering Research Center of Development and Utilization of Food and Drug Homologous Resources, Ministry of Education, Yunnan Agricultural University, 425 Fengyuan Road, Kunming 650201, China

**Keywords:** degradable film, walnut protein isolate, cellulose nanofiber, calcium cross-linking, curcumin

## Abstract

There is an inadequate supply of environmentally friendly, versatile disposable packaging films for protecting various food items. Herein, a series of walnut protein isolate (WPI)-based bioactive films, including WPI (W) film, cellulose nanofiber (CNF)-reinforced (WN) film, Ca^2+^-cross-linked WN (WNG) film, and curcumin-loaded WNG (WNGJ) film, were successfully fabricated. WNG showed the best properties in terms of elongation at break (53.47% ± 4.94%) and hydrophobicity (water contact angle of 91.94° ± 1.71°). WNGJ film showed the best properties in terms of antioxidant and antibacterial activities. Compared to the group without film protection, exterior quality, weight loss, softening, pH decreasing, and solids loss of strawberries were delayed around 1–3 d by each package, indicating the improvement in freshness. All bioactive films were more effectively at reducing the juice loss (improved 16–50%) and the spoilage of strawberries than the commercial film at room temperature over 5 days. Among them, WNGJ showed the best properties in terms of maintaining the firmness, acidity, and total soluble solids of strawberries. The films with tunable hydrophobicity properties and bioactive functions possess considerable potential usage in maintaining the freshness of perishable fruit.

## 1. Introduction

Currently, 430 million tons of plastic are produced globally each year, among which single-use plastics (SUPs) account for more than two-thirds [1]. Plastic food packaging is durable, lightweight and strong, with good mechanical properties and chemical resistance [2]. Biodegradable plastics currently account for around 0.5% of the total global production, while only around 25% of the plastic packaging is recycled, including single-use plastic food and drink containers [3,4]. However, the vast majority of plastic packaging materials are non-biodegradable in nature, and most end up being incinerated or landfilled, which result in large quantities of toxic gases (phenols, hydrogen chloride, carbon monoxide, sulfides, etc.), posing serious threats to both human health and the environment [5]. There is an urgent need for the development of diverse biodegradable packaging alternatives to meet various requirements of sustainable food packaging.

Recently, versatile packaging films became popular in food packaging for controlling mechanical injury, microbial contamination, moisture loss and lipid oxidation in substituting the conventional synthetic polymer [6]. Multiple biopolymers, such as natural edible polysaccharides (i.e., starch, κ-carrageenan, chitosan and cellulose derivatives) and plant proteins, especially water-insolubles (i.e., zein and soy protein), are used as excellent raw materials for multifunctional films due to their biodegradability, renewability, compatibility with biological systems, and safety [7,8,9]. Plant proteins derived from oil production showed superior film-forming capacity by controlled self-assembly [8]. Walnut protein isolate (WPI), a by-product of walnut oil processing, contains 70% water-insoluble glutenin including legumin B-like, 11S globulin-like, 11S globulin seed storage protein Jug r 4, and 11S globulin seed storage protein 2-like [10,11]. The glutenin of WPI possesses the ability to stabilize the air–water interface due to the formation of the solid-like interfacial film with a high elastic modulus (35–45 mN·m^−1^) [11]. Compared with soy protein or pea protein, the spatial conformation of WPI was more stable in fluctuating pH and temperature environments due to higher disulfide bond content [12], which offers greater potential for the subsequent modification and functional regulation of walnut protein-based package films. Another advantage is that WPI, as a film material, consumes less ethanol than zein [13,14]. However, applications of biopolymers in the packaging industry are limited by their low mechanical properties and high water and oxygen permeability due to the number of hydroxyl groups present in its structure. The film, which was constructed using a single protein and curcumin, showed limited improvement in hydrophobicity and reduced tensile strength, which compromised its overall performance [15].

Nanocellulose was used to improve both the mechanical properties and permeability properties of coating and packaging materials in food industry [16]. Films made from CNF exhibit extremely high air and oxygen barrier properties but possess high hydrophilicity [16]. When the content of cellulose nanofiber (CNF) increased from 2% to 4%, the tensile stress of the eucommia gum–CNF-based film was enhanced from 13.7 MPa to 15.7 MPa [17]. Meanwhile, the breaking elongation and Young’s modulus enhanced from 365.8% to 378.8% and from 89.0 to 120.1 MPa, respectively. The tensile strength of the CNF-based composite films with pullulan polysaccharide and pectin significantly increased, indicating enhanced interfacial bonding between the polymer matrix [18]. Although the oxygen barrier properties of nanocellulose films are competitive with the currently available commercial films made from synthetic polymers, their water vapor barrier remains low mainly because of the strong hydrophilic nature of the nanocellulose [19].

The complexes of protein and polysaccharide employed as the basic material for food packaging films have received more attention, and could achieve bespoke functions. Cellulose nanofiber or its derivatives are combined with other polymers to produce films that exhibited enhanced stability, augmented physical and mechanical properties, and customized packaging functions. Metal ion cross-linking typically occurs between two oppositely charged molecules, or between certain functional groups (such as hydroxyl or carboxyl groups) and multivalent metal ions [6]. Cellulose/lignin composite membranes prepared by mixing nanolignin and nanocellulose and impregnating them in a metal ion solution [20]. The Ca^2+^-cross-linked composite film exhibited the highest tensile strength (223.8 MPa), which surpassed the commercial petroleum-based plastics. Metal ion cross-linking between proteins and nanocellulose were a promising strategy to improve the properties of these composite biofilms. Furthermore, the bioactive substances incorporated in the composite biofilms, such as essential oil, anthocyanin, shikonin, thymol and curcumin, etc., achieve multiple functions. Among them, curcumin, (1,7-bis-(4-hydroxy-3-methoxyphenyl)-1,6-heptadiene-3,5-dione) a naturally occurring lipophilic pigment, was used as bioactive additive to enhance the functional properties of CNF-based films [9]. The antioxidant activity of curcumin was significantly enhanced by complexing with whey protein fibrils and chitosan [21]. The KGM-based film loaded with curcumin–Ca^2+^ chelate exhibited favorable thermal stability, mechanical properties, and antioxidant and antibacterial activities [22]. Thus, the multicomponent combination (the combined use of WPI, CNF, calcium, and curcumin additions) will achieve simultaneous improvements in structural stability and long-term active functions, offering a new formulation strategy for biopolymer-based active packaging films.

The hypothesis of this study was that the combination of WPI with CNF would produce a stable and biocompatible film matrix. Moreover, calcium ion cross-linking with curcumin loading would enhance the hydrophobicity, flexibility, antioxidant and antibacterial properties of the composite films, thereby facilitating the preservation of fruits. Therefore, new single-use packaging films were developed via the casting method by CNF or CNF combined with WPI as the main matrix, modifying the matrix through Ca^2+^ cross-linking and further curcumin addition. The cross-sectional and surface microstructures, physicochemical characteristics and functional qualities (antioxidant and antibacterial activities) of the films were assessed. Strawberry is a highly perishable fruit and typically keeps for 2 to 3 days at room temperature [23]. The preservation performance was evaluated by packaging of fresh strawberries. The composite films with tunable hydrophobic properties and bioactive functions possess considerable potential usage in maintaining the freshness of perishable fruit.

## 2. Materials and Methods

### 2.1. Materials

Walnut protein isolate (WPI, 92.7% protein content (dry basis)) was purchased from Shaan’xi TOSO Biotechnology Co., Ltd. (Xi’an, China). Cellulose nanofibers (CNF, length of 5–10 μm, diameter of 5–10 nm and carboxylate content of 1.80 mmol/g) at 1.2% wt/wt aqueous solution was purchased from Woodelfbio biotech Co., Ltd. (Tianjin, China). Anhydrous calcium chloride (CaCl_2_, 96%) was purchased from Macklin Technology Co., Ltd. (Shanghai, China). Glacial acetic acid (purity ≥ 98%) and glycerol (purity ≥ 99%) was purchased from Damao chemical reagent Co., Ltd. (Tianjin, China). Both 2, 2-diphenyl-1-picrylhydrazyl (DPPH) and 2, 2′-Azino-bis-3-ethylbenzothiazoline-6-sulfonic acid (ABTS) were obtained from Yuanye Biotechnology Co., Ltd. (Shanghai, China). All other reagents were of analytical grade. *Staphylococcus aureus* (*S. aureus*) and *Escherichia coli* (*E. coli*) cells for antibacterial assay were obtained from Qingke Biotechnology (Beijing, China). Strawberries were purchased from a fruit store (Kunming, China). Polyvinyl chloride (PVC) film was purchased from the Shanggou supermarket (Kunming, China). The soil was excavated from the farmland of the Yunnan Agricultural University (Kunming, China).

### 2.2. Preparation of Films

Four films were prepared by the casting method based on the preliminary experiment. The CNF dosage is the optimal concentration for forming a uniform film with certain mechanical support. CaCl_2_ concentration was adjusted to maintain film flexibility and prevent matrix aggregation. The dosage of curcumin is selected to achieve a balance between active functional performance and film-forming uniformity, thereby avoiding excessive curcumin precipitation and film opacity. The formulation of the composite films is shown in Table 1. The preparation steps for each film are as follows. (1) Firstly, WPI (1.0 g) was dispersed in distilled water (70 mL) by magnetic stirring for 30 min at 80 °C in a water batch. A portion of glacial acetic acid (5 mL) was added in the dispersion with continuous stirring for another 30 min at 80 °C. After centrifugation (3000 rpm, 5 min), the supernatant was obtained and poured into a beaker. Ethanol (5 mL) was incorporated in the solution. Then, the mixture of glycerol (0.6 g) and distilled water (10 mL) was added in the beaker. After magnetically stirring for 30 min, a film-forming solution (named W) was obtained. (2) WPI (1.0 g) and 0.6 wt% CNF aqueous solution (0.5 g) were dispersed in distilled water (70 mL) by magnetic stirring for 30 min. The subsequent steps were the same as those in (1). The resulting film-forming solution (named WN) was obtained. (3) WPI (1.0 g) and 0.6 wt% CNF aqueous solution (0.5 g) were dispersed in distilled water (70 mL) by magnetic stirring for 30 min. A portion of glacial acetic acid (5 mL, containing 0.1 g calcium chloride) was added in the dispersion with continuously stirring for another 30 min at 80 °C. The subsequent steps were the same as those in (2). The resulting film-forming solution (WNG) was obtained. (4) followed the process of (3). After centrifugation, 5 mL ethanol (containing 4 mg curcumin) was added into the supernatant with stirring at 80 °C. The film-forming solution was called WNGJ. All film-forming solutions (W, WN, WNG and WNGJ) were subjected to ultrasonic treatment (30 min, 600 W) to eliminate the foam. Each of the film-forming solution (20 mL) was then poured into a Petri dish and placed in a drying cabinet (DHG-2150B, Zhengzhou Shengyuan Instrument Co., Ltd., Zhengzhou, China) at 40 °C for 14 h. Finally, a thin film was formed and removed from the dish.

### 2.3. Structural Characterization of Films

The chemical structure of the films was measured by a Fourier transform infrared spectrometer (iS20, Thermo Scientific Nicolet, Waltham, MA, USA). The scanning wavelength range was 400–4000 cm^−1^ with 32 scans and a resolution of 4 cm^−1^.

The crystallinity of the films was characterized by X-ray diffractometer (XRD, Miniflex 600, Rigaku Corporation, Tokyo, Japan). The scan range (2θ) was 5–80° at the speed of 2°/min. The light source was CuKα radiation with the tube voltage of 40 kV and the current of 15 mA.

The microscopic morphologies of the surface and cross-section of the films were observed on a scanning electron microscopy (ZEISS GeminiSEM 300, Carl Zeiss Microscopy, Oberkochen, Germany). The samples were freeze-fractured in liquid nitrogen, sprayed with gold, and placed on a 6 mm diameter film disk. The voltage was set to 3.0 kV.

### 2.4. Characterization of Thickness, Moisture Content, Color and Opacity of the Films

The thickness of the film was measured using a portable digital micrometer (130-01-850, Qinghai Measuring Tools, Xining, China) with an accuracy of 0.001 mm. Each film was measured at 10 different positions.

The moisture content of the film was determined using a rapid moisture analyzer (HS153, Mettler Toledo, Greifensee, Switzerland). The film was weighed before and after drying at 105 °C until constant weight. The moisture content was recorded.

The chromaticity of the films (40 mm × 40 mm) was characterized using a CM-5 colorimeter (Konica Minota, Tokyo, Japan) with the parameters a* (greenness/redness), b* (blueness/yellowness), and L* (whiteness). Five measurements were conducted on the surface of each sample. The transmittance of the film (40 mm × 10 mm) was measured using a 756 spectrophotometer (Jinghua Technology, Shanghai, China) within the range of 200 nm to 800 nm. The opacity of the film was calculated using Formula (1):

(1) $$\mathrm{Opacity}=\frac{A_{600}}{X},$$

where *A*_600_ was the absorbance of the film at 600 nm, while *X* was the thickness of the film (mm), which was kept nearly ideal for all four tested films (around 50.4 to 60.6 μm).

### 2.5. Mechanical Properties, Water Contact Angle and Water Vapor Permeability of the Films

All composite films were stored in a constant temperature and humidity chamber (25 °C, relative humidity of 45%) for 48 h prior to testing their mechanical properties, water contact angle and water vapor permeability.

The mechanical properties of the films were determined using an electronic universal testing machine (CMT4503, MTS Systems Corporation, Eden Prairie, MN, USA), including tensile strength (TS), Young’s modulus (YM), and elongation at break (EAB). The tested film was cut into strips (10 mm × 50 mm). The initial gauge length and test speed were set to 30 mm and 1 mm/s, respectively. Each measurement underwent three tests.

The water contact angle of the film was measured using a water contact angle meter (SDC-350KS, Dongguan Shengding Precision Instrument Co., Ltd., Dongguan, China). A sample (2 mm × 2 mm) was placed on the contact angle testing platform. Then, a drop of deionized water (2 μL) was deposited onto the thin film surface using a high-precision injector. A high-speed camera captured images of the droplet. The droplet’s contour was numerically solved and fitted to the Laplace–Young equation to determine the contact angle. Three measurements were performed for each sample.

The water vapor permeability (WVP) of the film was determined using a gravimetric method [24]. A 10 mL glass vial with mouth diameter of 28 mm and depth of 57 mm was capped with the film sample. The vial contained known weights of anhydrous calcium chloride pellets. Then, each vial was weighed and placed in a desiccator containing 5 mL distilled water and kept at 35 °C. The vials were weighed every 12 h until constant weight was reached. The WVP was calculated with Formula (2):

(2) $$\mathrm{WVP}(g/m*h*\mathrm{Pa})=\frac{\Delta m\times D}{S\times T\times \Delta P},$$

where ∆*m* was the weight change of the vial (g), *D* was the thickness of the film, *S* was the exposed area of the film (m^2^), *T* was the time duration (h) of weight change, and ∆*P* was the vapor partial pressure (5626.7 Pa at 35 °C).

### 2.6. Antioxidant Activity of the Films

The antioxidant activity of the films was evaluated by DPPH and ABTS radical scavenging assays. A film sample (0.02 g) was cut into fragments and dissolved in ethanol solution (5 mL, 95%) with stirring at 50 °C for 2 h. The film solution (2 mL) was mixed with 2 mL DPPH solution (0.1 mmol/L) and incubated at room temperature for 0.5 h in dark. Ethanol solution (95%) was employed as the blank sample and the solvent. The absorbance of the sample (200 μL) at 517 nm was measured using a multifunctional microplate reader (Synergy H1, Agilent Technologies, Santa Clara, CA, USA). The DPPH radical scavenging rate (%) was calculated using Formula (3).

(3) $$\mathrm{DPPH}\left(\%\right)=(1-\frac{A_{s}-A_{b}}{A_{c}})\times 100\%,$$

where *A*_s_ denoted the absorbance value of the sample, *A*_b_ denoted the absorbance value of the DPPH solvent, *A*_c_ denoted the blank sample.

For the ABTS assay [25], mix 2 mL of ABTS solution (7.4 mM) with 2 mL of potassium persulfate solution (2.6 mM) and allow the mixture to react at room temperature in the dark for 12 to 16 h. Subsequently, dilute the ABTS solution with ethanol to achieve an absorbance of 0.7 ± 0.02 at 734 nm. A film fragment (0.1 g) was dissolved in ethanol solution (20 mL, 50%) with stirring at room temperature. The film solution (1 mL) was mixed with the ABTS solution (2 mL). The absorbance was measured at 734 nm after 6 min of reaction. The ABTS radical scavenging rate (%) was calculated using Formula (4).

(4) $$\mathrm{ABTS}\left(\%\right)=\frac{A-A_{i}}{A}\times 100\%,$$

where *A* was the absorbance value of the diluted ABTS solution, *A_i_* was the absorbance value of the film sample.

### 2.7. Antibacterial Activity of the Films

Antibacterial activities against *E. coli* and *S. aureus* were evaluated using the turbidity method [26], with slight modifications. Two bacteria were incubated in LB broth at 37 °C for 24 h at 150 rpm to reach the logarithmic growth phase (~10^7^ CFU/mL).

Film fragments (1 mm × 1 mm, 0.8 g) were subjected to UV-sterilizing for 30 min and added into 10 mL of the bacterial suspension or LB broth at 37 °C. Then, each mixture was shaken at 150 rpm for 6 h. The absorbance at 600 nm was measured. The inhibition rate was determined as follows (Formula (5)):

(5) $$\mathrm{Antibacterial}\;\mathrm{ratio}(\%)=(1-\frac{A_{1}-A_{2}}{A_{0}})\times 100,$$

where *A*_0_ is the absorbance of bacterial suspension, *A*_1_ is the absorbance of bacterial suspension with film sample, and *A*_2_ is the absorbance of LB broth with film sample.

### 2.8. Biodegradation of the Films

A preliminary soil-disintegration test was conducted based on a previous method in soil [27]. The tested soil was mountain red soil collected from the experimental farm of the Yunnan Agricultural University (Kunming, China). The soil exhibits an acidic pH range of 5.46–6.47, characterized by a clay loam texture. Each piece of film (2 cm × 2 cm) was buried in soil for one month. During the experiment period, the morphology change of the sample was observed and recorded daily.

### 2.9. Application in Strawberry Preservation

The film was used for strawberry packaging. Fresh strawberries (*Fragaria × ananassa* Duch. cv. Akihime) were selected at the commercially mature stage (70–80% surface redness) with similar size and no mechanical damage or disease for the experiment. All strawberries used in the experiment came from the same batch and were divided into six groups. Each group consisted of 45 strawberries. The control group was without packaging (bare). The positive group was wrapped in polyvinyl chloride film (PVC). The remaining four groups were packaged with W, WN, WNG, and WNGJ films, respectively. All samples were stored at room temperature (18–22 °C, relative humidity of 40–55%). The experiment was repeated twice with independent batches.

*Appearance and weight loss*. The appearance and combined weight of the fruit and the packaging were recorded daily. The percentage weight loss (%) of the strawberries was calculated by comparing their weight after 1, 2, 3, 4, and 5 days of storage with their initial weight.

*Firmness.* The firmness of the fruits was measured by a handheld penetrometer (GY-4, Yueqing Aidebao Instrument Co., Ltd., Yueqing, China) equipped with an 8 mm diameter probe. The maximum force (N) was recorded and considered as the firmness of the fruit. Each group measured three replicate samples.

*pH values.* The pH value of strawberries was measured using a pH meter (EL20, Mettler Toledo, Greifensee, Switzerland). In short, strawberries were crushed, then centrifuged using a low-speed centrifuge (LC-4014, Anhui Zhongke Zhongjia Scientific Instrument, Hefei, China). The supernatant was collected and analyzed.

*Soluble solid content.* The strawberries were squeezed to obtain juice. One drop of the juice was placed on a handheld refractometer to record the soluble solids content. Each group was measured three replicate samples.

### 2.10. Statistical Analysis

Each group of films was prepared in triplicate. All replicates in film characterization are derived from independently prepared batches. Data were analyzed using SPSS 29.0 and presented as the mean ± SEM of three replicates. The significance of differences was assessed using analysis of variance (ANOVA) and Tukey’s multiple comparison test (*p* <0.05).

## 3. Results and Discussion

### 3.1. Appearance, Color Parameters, Transparency and Thickness of the Films

**Figure 1:**
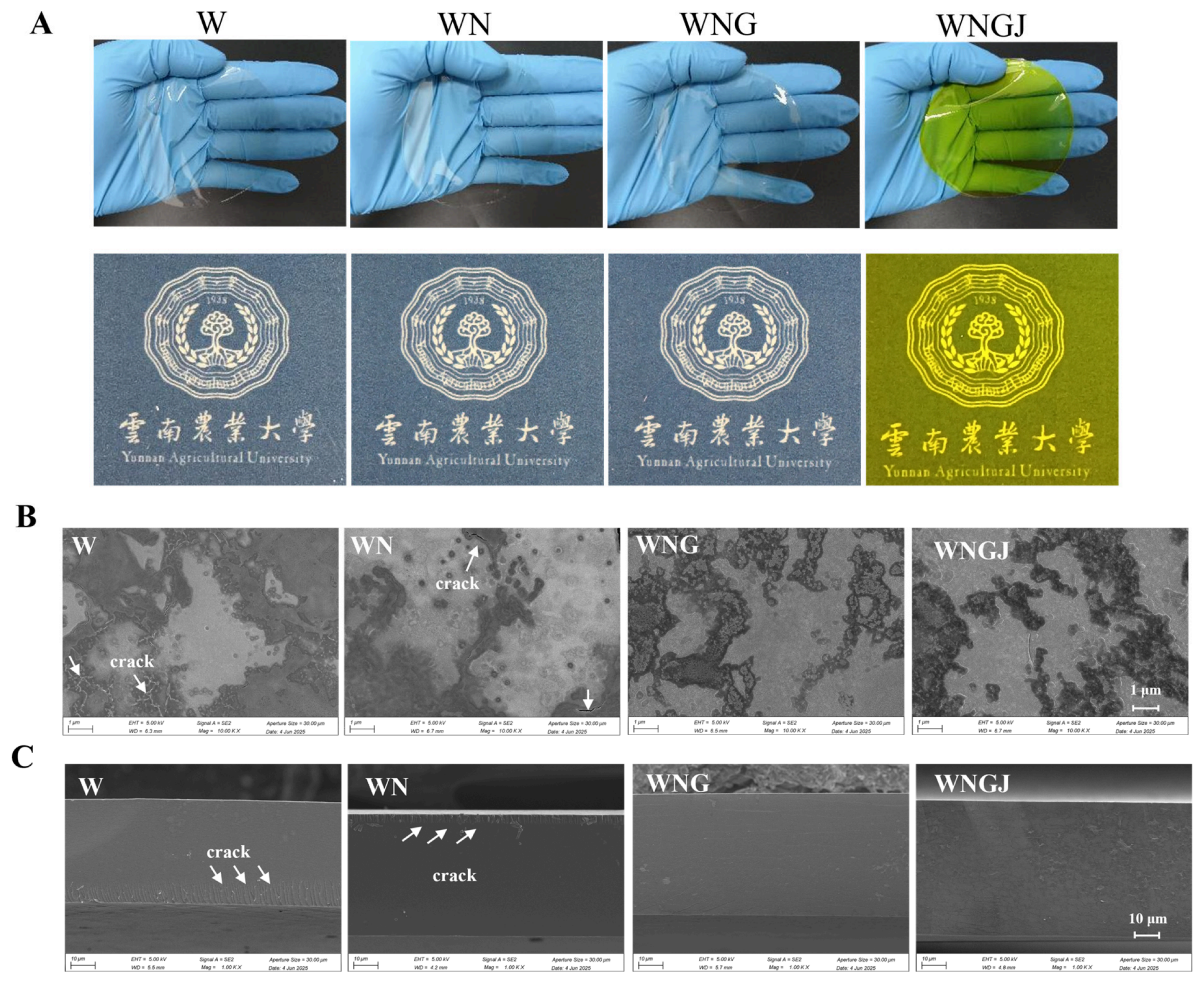
Appearance of the composite films (**A**). SEM images of the surface (**B**) and cross-section (**C**) of the composite films.

The visual appearance of the cling film, particularly with regard to color and transparency, plays a crucial role in consumer acceptance. The visual appearances of the films are shown in Figure 1A. The W, WN and WNG films were almost transparent, which was an ideal appearance for consumers, while the WNGJ film exhibited a luminous yellow, which was attributed to the color of curcumin. Accordingly, the color parameters of the films including L* (brightness), a* (red–green value), and b* (yellow–blue value) are shown in Table 2. The color parameters of the W, WN and WNG films showed no significant difference (*p* > 0.05) with L* value near 90 and both a* and b* values around 0, which verified the transparency of the films. Compared to the W, WN and WNG films, the L* and a* values of the WNGJ film decreased significantly (*p* < 0.05), while the b* value of the film increased markedly (*p* < 0.05), indicating the bright yellow color of the film. This was due to the incorporation of curcumin. A similar change was observed in carboxymethyl cellulose-based film with the addition of curcumin [28].

The transmittance of a film is related to its matrix, thickness, and color. Compared to the W film (1.27 ± 0.03 Abs*mm^−1^, Table 2), WN exhibited the highest opacity value (1.66 ± 0.04 Abs*mm^−1^), indicating that the addition of CNF partially impaired the light transmission properties of the film. As reported previously, the transmittance of bionanocomposite films prepared with agar and paper-mulberry pulp nanocellulose was decreased linearly with the increase in nanocellulose content [29]. However, after Ca^2+^ cross-linking between WPI and CNF, the opacity of the WNG film decreased due to the reduction in the CNF’s light-blocking effect, thereby significantly enhancing the light transmittance of the film. Compared to WNG films, the opacity of the WNGJ film increased a little due to the light absorption capacity of curcumin.

### 3.2. Microstructure of the Films

The microstructure of the WPI-based films was investigated by observation of both the surface (Figure 1B) and the cross-sections (Figure 1C). As shown in Figure 1B, numerous cracks appeared on the surface of the W film (denoted by arrows), indicating an inhomogenous structure. Compared to W films, the number of cracks on the surface of WN films was significantly reduced, indicating a more uniform microstructure. New aggregates were observed on the surface of the WNG film with no distinct cracks, indicating a uniform film structure. After adding curcumin, the aggregate structure on the surface of the WNGJ film became more pronounced, although no cracks were observed, which indicated the structural integrity of the composite film was maintained. The aggregate distribution in WNG and WNGJ films was different, likely due to the uneven distribution of calcium ions. Calcium ions may selectively cross-link with the hydroxyl groups of CNF, the carboxyl groups of WPI, or the phenolic hydroxyl groups of curcumin. As shown in Figure 1C, unilateral wrinkling was observed in the cross-sections of both W and WN films, which may be attributed to excessive dehydration of the surface exposed to hot air during the drying process. In contrast, the cross-sections of WNG and WNGJ films exhibited a denser microstructure without cracks. Compared to the WNG film, a more coarse aggregate was observed in the cross-section of the WNGJ film, which was attributed to precipitation of curcumin crystals [22].

### 3.3. FT-IR Spectra and XRD Patterns of the Films

**Figure 2:**
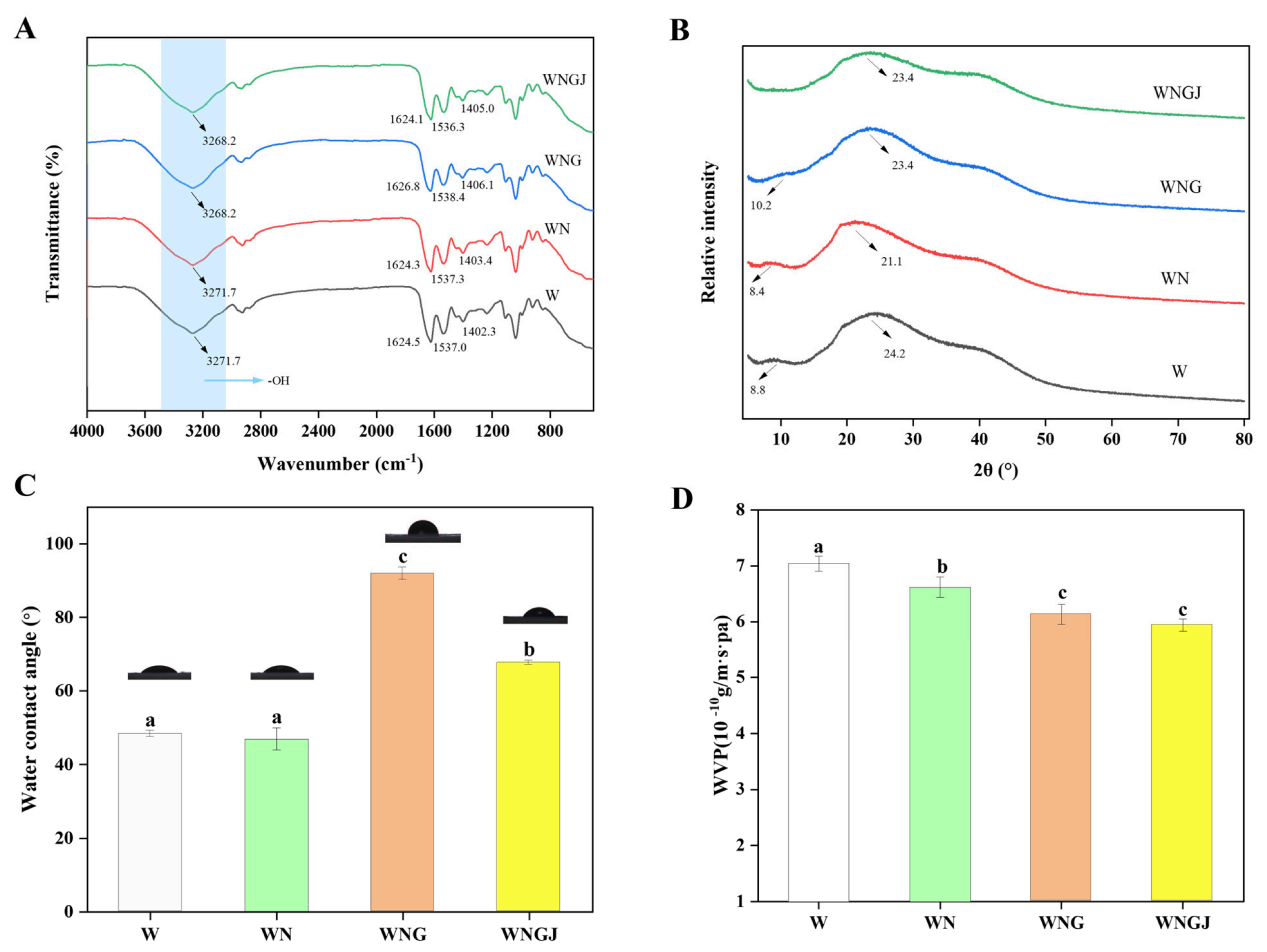
Fourier transform infrared spectroscopy (FT-IR) spectra (**A**) and X-ray diffraction patterns (**B**) of the composite films. Water contact angle (**C**) and water vapor permeability (**D**) of the composite films. Different letters (a–c) indicated significant differences (*p* < 0.05).

*FT-IR spectra.* The molecular interactions among different components within the WPI films were reflected by FT-IR spectroscopy. The FT-IR spectra of the films are shown in Figure 2A. Three distinctive peaks at 1624.5 and 1537.0 cm^−1^ were observed in the FT-IR spectrum of the W film, corresponding to C=O stretching (amide I band) and bond (amide II band), respectively. A strong and broad peak at around 3271.7 cm^−1^ was observed in the FT-IR spectra of W and WN films reflecting the stretching vibration of the -OH group. In contrast, a red shift (3268.2 cm^−1^) was observed in the spectra of WNG and WNGJ films, indicating that hydroxyl groups participated in both ion cross-linking and metal coordination. Compared to the W film, no new peaks were observed in the FT-IR spectrum of WN film. Liu et al. [30] reported similar findings that the FT-IR spectrum of the guar gum and sodium alginate composite film with carboxylated cellulose nanofibers (CNF) addition resembled that of the film without CNF addition. The peak at 1624.5–1624.3 cm^−1^ corresponded to C=O stretching vibrations, the peak at 1537.0–1537.3 cm^−1^ corresponded to N-H bending vibrations and C-N stretching vibrations, and the peak at 1402.3–1406.1 cm^−1^ corresponded to -OH bending vibrations. Compared to WN film, WNG film exhibited shifts from 1624.3 cm^−1^ to 1626.8 cm^−1^, from 1537.3 cm^−1^ to 1538.4 cm^−1^, and from 1403.4 cm^−1^ to 1406.1 cm^−1^. This indicated that the incorporation of calcium ions caused the peak positions to shift toward higher wavenumbers. After the curcumin–metal complex was added to the konjac glucan film, the -OH vibrational peak also shifted [22].

Upon further addition of curcumin, the peak position shifted toward lower wavenumbers. Specifically, compared to WNG film, the WNGJ film exhibited peak shifts from 1626.8 cm^−1^ to 1624.1 cm^−1^, from 1538.4 cm^−1^ to 1536.3 cm^−1^, and from 1406.1 cm^−1^ to 1405.0 cm^−1^. Interestingly, after adding calcium chloride, the peak position of the WNG film shifted toward higher wavenumbers. Upon further addition of curcumin, the peak position of the WNGJ film shifted back toward the original lower wavenumber region. This trend aligned with the thin film’s hydrophilicity–hydrophobicity changes: calcium chloride increased hydrophobicity, while curcumin reduced it. This possibly suggested a potential correlation between FTIR peak shifts and the thin film’s hydrophilic–hydrophobic properties. Ion cross-linking occurred through ionic bonds or electrostatic interactions, rather than through the formation of other new chemical bonds [6].

*XRD patterns*. As shown in Figure 2B, the XRD pattern exhibited two diffraction peaks in the ranges of 8.4–10.2° and 21.1–24.2°. The 2θ angles of the W, WN, and WNG films were 8.8°, 8.4°, and 10.2°, respectively, with corresponding intermolecular distances of 10.04 Å, 10.52 Å, and 8.66 Å. Compared to W and WN films, the WNG film exhibited reduced diffraction peak area at 10.2 ° and decreased intermolecular spacing, indicated successful cross-linking of CNF and WPI by calcium ions. The disappearance of diffraction peaks around the 8.4–10.2° region in WNGJ films indicated that curcumin addition altered the structure of Ca^2+^-cross-linked CNF-WPI films. All films exhibited broad peaks around 23.4°, indicating an amorphous structure [31]. Compared to the amorphous peak corresponding to 24.2 ° in the W film, the amorphous peak in the WN film shifts toward lower angles, exhibiting a broad peak at 21.1°. This indicated that CNF influenced the aggregation structure of the W film, resulting in a more compact structure, which was consistent with the findings observed by SEM.

### 3.4. Mechanical Properties

Mechanical properties are critical parameters for food packaging films. The tensile strength, elongation at break (EAB), and Young’s modulus of the four films are shown in Table 3. The tensile strength of the W film was 6.10 ± 2.45 MPa. After incorporating CNF, both the tensile strength and EAB of the WN film decreased, indicating that CNF filling in the WPI-based film affected the molecular interactions. After calcium ion addition, the tensile strength of the WNG film dropped to 1.74 ± 0.45 MPa. However, the EAB of WNG film increased by 58.2% compared to W films, indicating that calcium ion cross-linking significantly enhanced the flexibility of the films. After curcumin addition, the WNGJ film exhibited improved mechanical properties compared to the WNG film, with comparable tensile strength and EAB values (*p* > 0.05) to W film. It was believed that the EAB value of a film was related to the fluidity of the polymer chains [32]. With incorporation of CNF, the fluidity of the WPI chains decreased, thereby decreasing EAB. Subsequently, the increase in EAB value for the WNG film could be attributed to calcium ion cross-linking between WPI and CNF enhancing the movable chain length. But the addition of curcumin in the film-forming matrix affected the effective movement of cross-linking chain.

### 3.5. Water Contact Angle, Moisture Content and Water Vapor Permeability

*Water contact angle.* The water contact angle reflected the hydrophilic or hydrophobic properties of food packaging material. As shown in Figure 2C, the water contact angles of W and WN films were about 47–48°, exhibiting their hydrophilic property. However, the Ca^2+^-induced cross-linking of carboxyl group of WPI or hydroxyl groups of CNF and WPI notably increased the water contact angle of WNG film to nearly 92°. The adjustment of chemical cross-linking of the film led to the decrease in hydrophilic groups (free hydroxyls originated from CNF) on the surface, which intensified the overall hydrophobic nature of the film. This stopped moisture from evaporating and microorganism penetration and ultimately helped to keep food fresh. Compared to the zein/sodium alginate composite film (water contact angle of 60°) [14], the WNG film demonstrated better hydrophobicity as similar plant protein-based films. Upon further incorporation of curcumin, the hydrophobicity of WNGJ film decreased slightly. It was attributed to the potential interaction between the carbonyl and hydroxyl groups in curcumin and the hydroxyl groups in WPI and CNF, which diminished the cross-linking degree of calcium ions with CNF and WPI. However, the hydrophobicity of WNGJ films remained significantly higher than that of W and WN films.

*Moisture content.* Moisture content is related to the total free space taken up by water molecules in the film. The relatively looser microstructure of the film resulted in higher available volume of free water, suggesting faster water transport rate [33]. As shown in Table 2, the moisture content of W film was the highest (24.41%), while the moisture content of WN, WNG and WNGJ films were significantly reduced (*p* < 0.05), indicating the addition of CNF or incorporation with Ca^2+^ or Ca^2+^ and curcumin reinforced the structure of W film. CNF filled the microscopic pore and interstices of the W film matrix, thereby reducing the encapsulation and binding sites between the film and water molecules. Since the calcium ion neutralized the negative ions (carboxyl) on CNF, it reduced the electrostatic repulsion between CNFs [34]. The cross-linked matrix formed by calcium ions and curcumin further intensified the physical barrier for the migration of water molecules.

*Water vapor permeability.* Water vapor permeability (WVP) is a key parameter for evaluating the barrier properties of food packaging films. As shown in Figure 2D, the WVP of the W film was 7.04 × 10^−10^ g/m·s Pa, which might be attributed to the loose structure of the W film with only a WPI matrix. The addition of CNF and the cross-linking with Ca^2+^ significantly decreased the WVP of the WNG film to WVP of 6.13 × 10^−10^ g/m s Pa. The three-dimensional structure constructed by CNF with WPI filling in the network strengthened the originally loose protein-based film structure, which enhanced the barrier against water vapor penetration. The barrier properties of agar-based composite films were enhanced by incorporating nanocellulose [35]. After incorporating curcumin in the matrix, the WVP of the WNGJ film remained unchanged. The addition of hydrophobic curcumin to the composite films made by bacterial cellulose nanofibers and gelatin resulted in a prolonged path for water molecules to cross through the films [36].

### 3.6. Biological Activities of the Films

**Figure 3:**
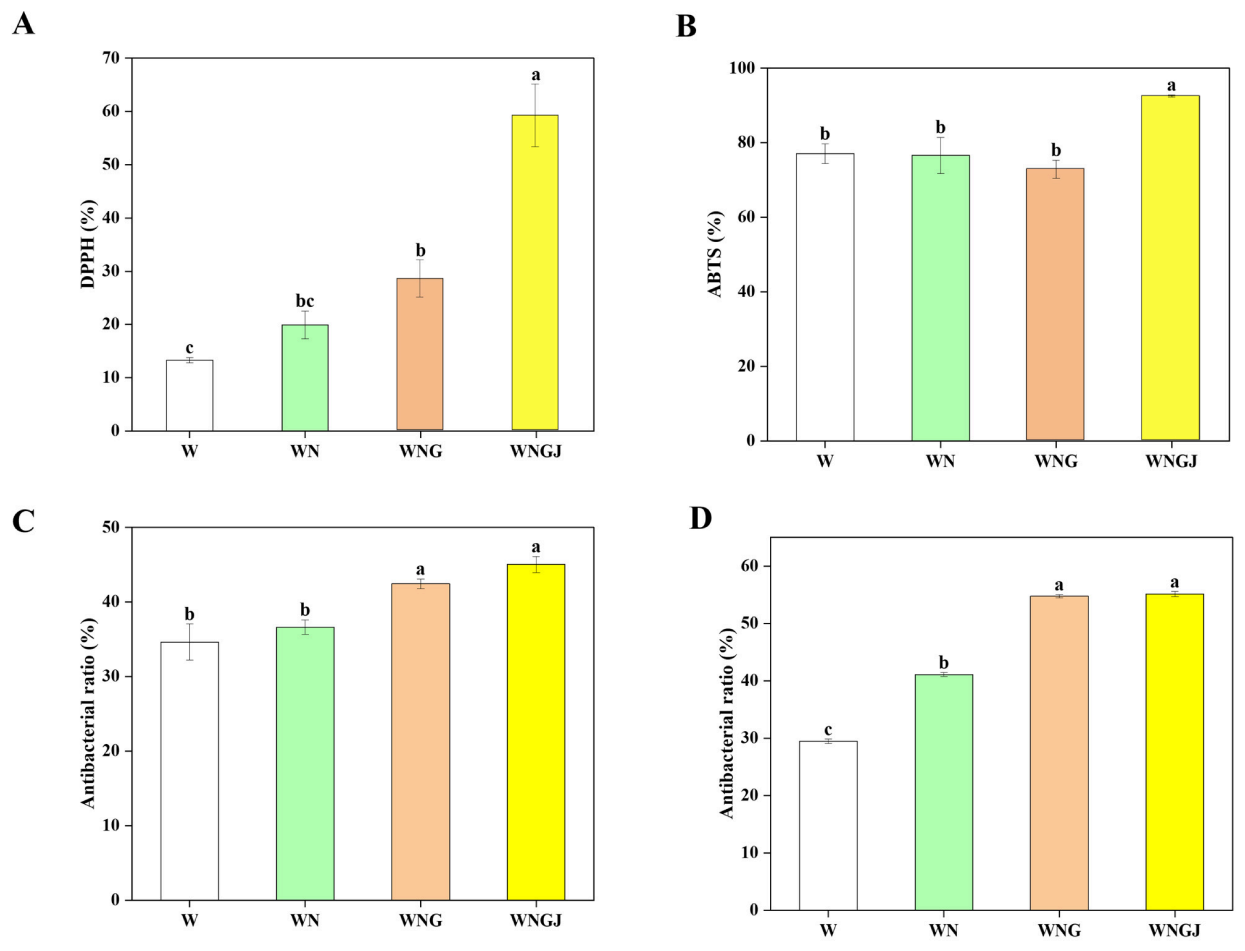
The DPPH (**A**) and ABTS (**B**) free radical scavenging capacities of composite films. The antibacterial activity of the films against *E. coli* (**C**) and *S. aureus* (**D**). Different letters (a–c) indicate significant differences (*p* < 0.05).

*Antioxidant activities.* Antioxidant activities reflected the availability and effectiveness of the fresh-keeping films in preventing the packaged food stuff from oxidative browning and caducity. The antioxidant capacity of the films was evaluated by DPPH and ABTS radical assays and the results are shown in Figure 3A,B. As shown in Figure 3A, all the tested films were capable of scavenging a certain amount of DPPH radicals. The scavenging rate of the W film was 13.3%, while the values of WN, WNG and WNGJ films were increased in order, with scavenging rates of 19.9%, 28.5%, and 59.2%, respectively. CNF was reported to possess certain antioxidant capabilities [37]. Incorporation of curcumin after Ca^2+^-cross-linking WPI and CNF, the antioxidant capacity of the film was further enhanced (*p* < 0.05). For the ABTS radical (Figure 3B), all films showed high scavenging capacity (above 70%). There was no significant difference among the ABTS radical scavenging rates of W, WN, and WNG films (*p* > 0.05). Among the four groups, WNGJ film exhibited the strongest scavenging capacity against ABTS radicals (92.6%) among the films. The highest antioxidant capacity of the WNGJ film was attributed to the curcumin component, on which the p-methoxy group was confirmed to have high oxidation activity [38].

*Antibacterial properties.* The inhibitory effect of the films on foodborne pathogens is a key indicator for evaluating their freshness-preserving performance. As shown in Figure 3C, the inhibition rate of W and WN films against *E. coli* was similar (34.62% and 36.61%, respectively), suggesting a limited inhibitory effect. This was consistent with the results reported by [39]; the addition of CNF does not significantly enhance the antibacterial abilities of the prepared films. Compared to the WN film, the antibacterial rate of the WNG film was increased to 42.43%, indicated that Ca^2+^ cross-linking enhanced the inhibitory effect of the film against *E. coli.* From Figure 3D, the inhibition rate of the WN film against *S. aureus* was significantly increased to 41.08% compared to the W film (29.43%), indicating that CNF addition enhanced the inhibitory effect of the film against *S. aureus*. *E. coli* is a Gram-negative bacterium with a thinner cell wall compared to *S. aureus*; therefore CNF and the further Ca^2+^ crossing effect may exert differing effects on the stability of its cell wall. After adding curcumin, the inhibition rates of WNGJ film against *E. coli* and *S. aureus* were comparable to WNG (without significant difference). The antibacterial efficacy of the curcumin loading was due to the disruption of the peptidoglycan layer within bacterial cell wall [15]. However, in this study, curcumin was trapped within the cross-linking membrane, making it difficult to dissolve rapidly. Compared to the W film, the inhibition ratio of WNG and WNGJ films was comparably increased by about 1.2–1.3 and 1.9 times.

### 3.7. Biodegradability of the Films

**Figure 4:**
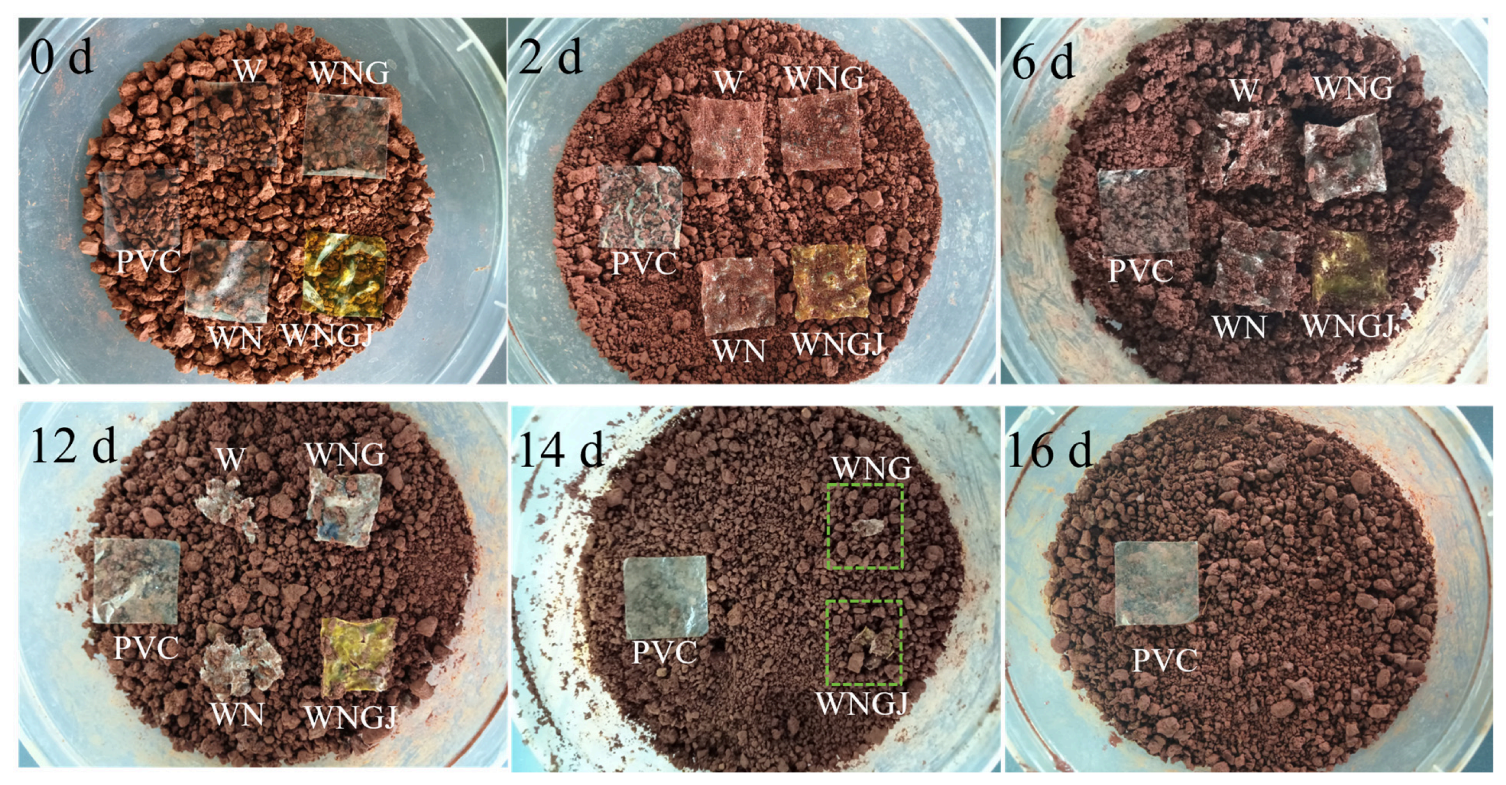
Biodegradability of the polyvinyl chloride (PVC) film (control) and the composite films in soil.

The eco-friendliness of food packaging films has gained substantial attention, which is often gauged by biodegradability in soil or water. The soil biodegradability of the WPI-based films was evaluated by observing the changes in film residues after being buried in natural soil. The results are shown in Figure 4 with commercial PVC cling film serving as the control. After burying in the soil for 6 d, W film occurred a fractional disintegration compared to the control. This was primarily attributed to the fact that proteins can be directly degraded into amino acids by soil microorganisms [40]. In contrast, other films merely showed wrinkling but remained intact, indicating the delayed degradation of the films by the CNF addition. After 12 days, all tested films with various formulae had undergone decomposition, except for the control. W and WN films completely degraded after being buried in the soil for 14 days, while the complete degradation of WNG and WNGJ films took 16 days. The results suggested that all films were degradable. The order of the remaining size of the films was as follows: W < WN < WNG < WNGJ, indicating that the Ca^2+^ cross-linking and curcumin addition delayed the degradation of WNG and WNGJ films. Among the tested films, the longest degradation of the WNGJ film was also attributed to the antibacterial properties of curcumin. External reinforcement and cross-linking enabled the usable period of the WPI film to be appropriately extended, which was of great significance for becoming a single-use preservation material.

### 3.8. Strawberry Preservation

**Figure 5:**
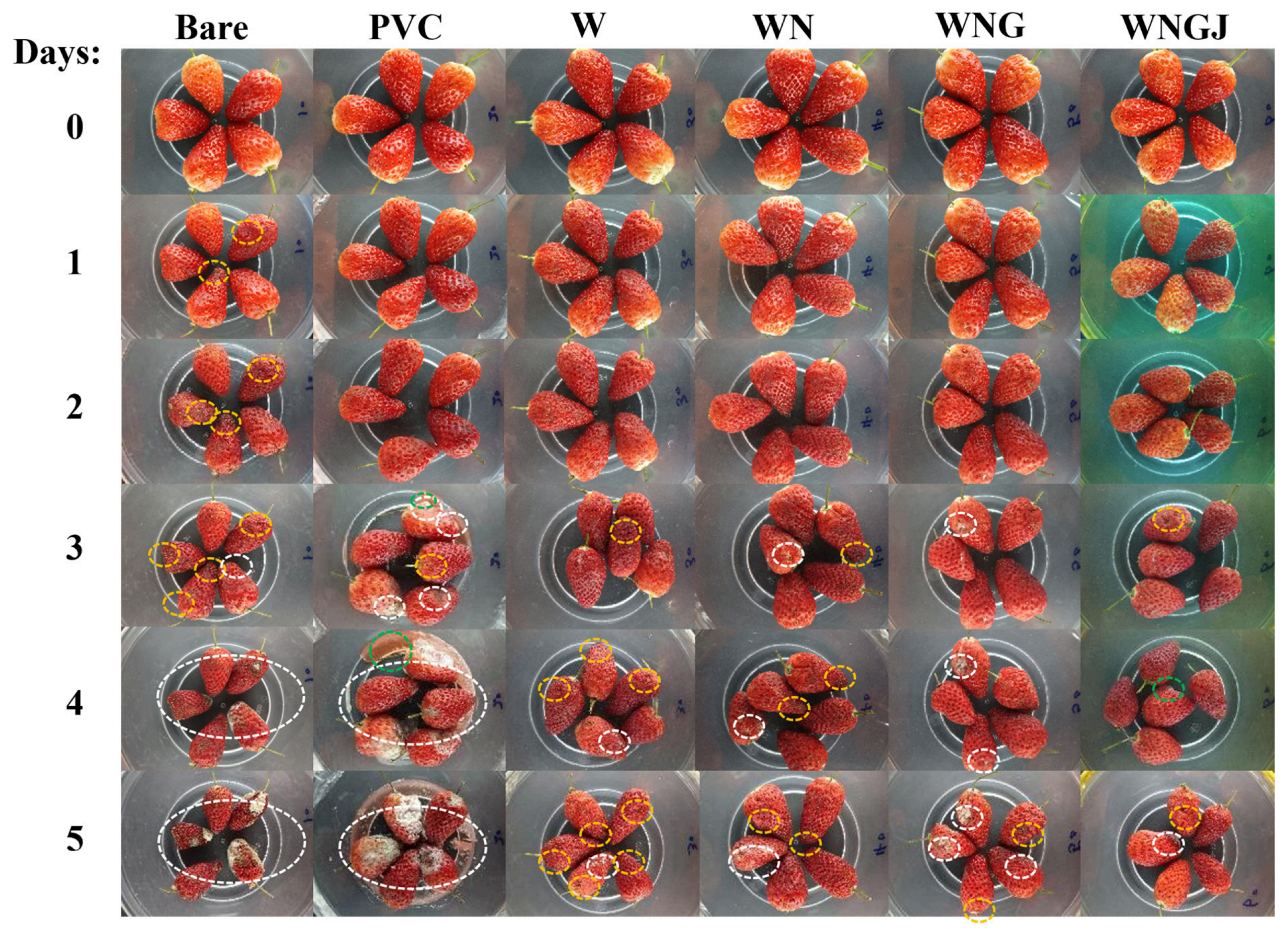
Changes in appearance of strawberries treated with the films during storage. White circles indicated mold, yellow circles indicated shrivel, and green circles indicated the leakage of strawberry juice.

**Figure 6:**
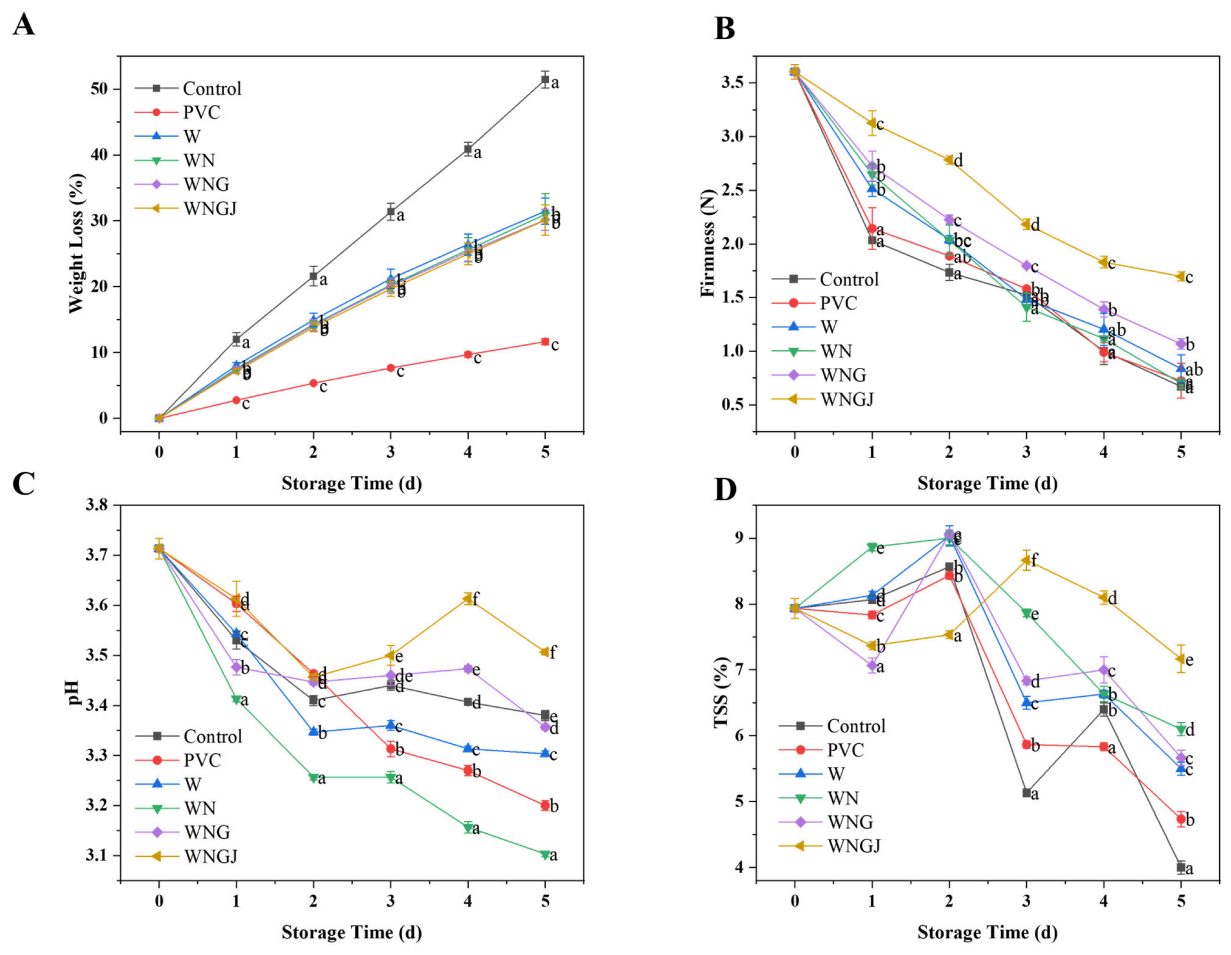
Weight loss (**A**), firmness (**B**), pH value (**C**) and total soluble solids (**D**) of strawberries treated with the films during storage. Different letters (a–e) indicate significant differences (*p* < 0.05).

Strawberries are highly perishable fruits, and extending their shelf life holds significant economic importance. The disposable packaging film is a commonly used material for preserving strawberries, and its large-scale application is one aspect that contributes to environmental pressure. Four WPI-based films were used to package fresh strawberries, and the results are shown in Figure 5 and Figure 6. As shown in Figure 5, the unpackaged strawberries (Bare) were significantly shrunk and darkened after being stored for 1 to 3 days. Three days later, the strawberries began to deteriorate. About 4 to 5 days later, the samples were completely inedible. The strawberries wrapped in PVC cling film were plump with attractive appearance within 2 days of storage. However, some parts of the surface of these strawberries began softening from the second day of storage. Then, almost all the strawberries began to rot, and the rotting process was extremely rapid. These results indicated that the commercially available cling film (PVC) possessed a good ability to prevent water loss of strawberries, but an overly humid environment accelerated the softening and spoilage of the samples. In contrast, strawberries with the protections of the W, WN, WNG, and WNGJ films showed no obvious shrinkage or black patches after 2 days of storage. After storage for 5 days, the spoilage degree of strawberries in the four treated groups were all lower than those in the PVC group, especially in the WNGJ group, although slight dehydration was observed in the treated groups (typical W group), which was less than the bare samples but observed more than the PVC-covered strawberries. Furthermore, no obvious softening or juice exudation were found in the four treated groups like in the PVC group.

The respiration and transpiration of fruits, along with the ethylene they release, significantly impact fruit quality [41]. As storage time increased, the weight loss of strawberries also gradually increased (Figure 6A). The weight loss of strawberries in the bare group was the highest (51.45% ± 1.27%), while the weight loss of those in PVC group was the lowest during the 5-day storage period. This indicated the excellent water impermeability of commercial cling films like PVC and PE [42]. The weight loss of strawberries was moderate among the W, WN, WNG, and WNGJ groups, which was significantly higher than that in PVC group but markedly lower than that of the bare samples. However, there was no significant difference among the treated groups.

Firmness is a key parameter for evaluating the freshness quality of strawberry. As shown in Figure 6B, the firmness of strawberries in each group gradually decreased with the storage time, indicating the softening of internal tissue. Cellulose nanofiber-based packaging films synergistically reinforced with lignin and tea polyphenols for strawberry preservation delayed the softening of strawberry [43]. Results showed that the firmness of strawberries in the bare and PVC groups were similar. Among the treated groups, only the firmness of strawberries in WNG and WNGJ groups were higher than the controls. This was due to the enhanced compactness of the film through calcium ion cross-linking, while the addition of curcumin further improved the antioxidant and antibacterial properties of the film.

The pH level of strawberries is a key factor influencing their flavor. Fresh strawberries release ethylene, which accelerates their ripening process, deepening their red color and lowering their pH level [41,44]. As shown in Figure 6C, the pH of strawberries across all groups exhibited an overall downward trend as the storage time was prolonged. For the bare group, the pH of the strawberries fell rapidly within the first two days, and then decreased slowly at day 4 and day 5. For PVC group, the pH decreasing lasted until the end, which was probably due to beneficial microorganism growth and reproduction under high-moisture conditions. As the storage time increased, the combined effects of moisture loss and organic acid decomposition possibly exerted a greater influence on the pH of strawberry, causing the pH to rise. The impact of microbial growth and reproduction on strawberry pH outweighed water loss and organic acid decomposition, which resulted in the accelerated reduction in the pH of strawberries. At the last 3 days, the pH of the sample in the WNG and WNGJ groups were higher than the PVC, reflecting the effectiveness in protecting strawberries from spoilage or decay.

Total soluble solids (TSS) indicated the dry matter content of the fruits. As shown in Figure 6D, the TSS of strawberries exhibited an overall declining trend, which was consistent with the weight loss. After 5-day storage, the TSS of all the treated groups (5.6–7.4%) was higher than the negative (Bare, 4.1%) and positive (PVC, 4.8%) controls. This demonstrated that all WPI-based films increased the retention rate of soluble solids in strawberries. Among them, the WNGJ film was the most effective (7.4%), which was attributed to the strong antioxidant properties of curcumin that prevented the post-harvest enzymatic deterioration of strawberries. Therefore, the composite films were a promising alternative to commercial disposable food preservation films, which effectively extended the shelf life of fruits.

## 4. Conclusions

Four composite films were constructed by the casting method using WPI and CNF as structural materials. To enhance the mechanical properties, and antioxidant and antibacterial activities of the composite film, Ca^2+^ cross-linking and curcumin loading were employed. The flexibility and hydrophobicity of the composite film was improved by the Ca^2+^ cross-linking, while the tensile strength of the film was decreased to some extent. Among the four different formulations, WNGJ films exhibited the strongest DPPH and ABTS radical scavenging activities (59.2% and 92.6%, respectively) and antibacterial effect against *E. coli* and *S. aureus* (130.04% and 187.25%, respectively, compared to the W film). The sequential additions of cellulose nanofibers, calcium chloride, and curcumin progressively reduced the water vapor transmission rate of the film. All films are degradable in soil. The spoilage, weight loss, pH decline and softening of strawberries were effectively delayed by the composite films, especially the WNGJ film. It provides a feasible green alternative to traditional plastic packaging materials and has broad application potential in sustainable food packaging industry.

In addition, the study has some limitations. First, this work only completed laboratory-scale preparation and short-term room-temperature fresh-keeping tests. The long-term storage stability of the film, industrial-scale film-forming adaptability, and actual commercial packaging application effect still need further verification. Second, the soil degradation test is only a preliminary qualitative exploration, and further dynamic degradation mechanism research has not been studied. In this research degradation performance of the film was only qualitatively evaluated, due to the high viscosity of the selected soil making it difficult to remove from the films. Quantitative mass loss of the film before and after degradation should be investigated in the future by measuring the weight loss using other soils.

## Figures and Tables

**Table 1:** The formulation of the composite films.

Films	Components							
	WPI (g)	CNF (g)	Calcium Chloride (g)	Curcumin (mg)	Glacial Acetic Acid (mL)	Ethanol (mL)	Glycerol (g)	Distilled Water (mL)
W	1.0	/	/	/	5	5	0.6	80
WN	1.0	0.5	/	/	5	5	0.6	80
WNG	1.0	0.5	0.10	/	5	5	0.6	80
WNGJ	1.0	0.5	0.10	4.0	5	5	0.6	80

**Table 2:** Color, thickness, opacity and water content of the composite films.

Film	Color Parameters			Thickness (μm)	Opacity (Abs*mm^−1^)	Water Content (%)
	L*	a*	b*			
W	96.65 ± 0.07 ^a^	−0.06 ± 0.10 ^a^	−0.11 ± 0.13 ^b^	50.4 ± 12.5 ^a^	1.27 ± 0.03 ^b^	24.41 ± 0.78 ^a^
WN	96.12 ± 0.09 ^a^	−0.07 ± 0.01 ^a^	−0.07 ± 0.13 ^b^	51.0 ± 17.2 ^a^	1.66 ± 0.04 ^a^	19.39 ± 0.55 ^b^
WNG	96.10 ± 0.06 ^a^	−0.22 ± 0.02 ^a^	0.11 ± 0.07 ^b^	57.4 ± 19.9 ^a^	1.06 ± 0.02 ^c^	20.02 ± 0.97 ^b^
WNGJ	93.31 ± 0.42 ^b^	−6.02 ± 0.34 ^b^	15.00 ± 0.78 ^a^	60.6 ± 13.7 ^a^	1.28 ± 0.05 ^b^	19.88 ± 0.30 ^b^

Note: Different letters within the same column represent significant difference (*p* < 0.05).

**Table 3:** Tensile strength, elongation at break, and Young’s modulus of the composite films.

Film	Tensile Strength (MPa)	Elongation at Break	Young’s Modulus (MPa)
W	6.10 ± 2.45 ^a^	33.79% ± 5.80% ^b^	78.22 ± 26.86 ^a^
WN	3.38 ± 0.51 ^ab^	21.02% ± 5.32% ^c^	24.73 ± 7.12 ^b^
WNG	1.74 ± 0.45 ^b^	53.47% ± 4.94% ^a^	25.71 ± 13.85 ^b^
WNGJ	4.01 ± 1.76 ^ab^	28.99% ± 1.46% ^bc^	46.63 ± 24.88 ^ab^

Note: Different letters within the same column represent significant difference (*p* < 0.05).

## Data Availability

The raw data supporting the conclusions of this article will be made available by the authors on request.
